# Pharmacogenomic-guided dosing of fluoropyrimidines beyond *DPYD*: time for a polygenic algorithm?

**DOI:** 10.3389/fphar.2023.1184523

**Published:** 2023-05-15

**Authors:** Anthi Maslarinou, Vangelis G. Manolopoulos, Georgia Ragia

**Affiliations:** ^1^ Laboratory of Pharmacology, Medical School, Democritus University of Thrace, Alexandroupolis, Greece; ^2^ Individualised Medicine and Pharmacological Research Solutions Center, Alexandroupolis, Greece; ^3^ Clinical Pharmacology Unit, Academic General Hospital of Alexandroupolis, Alexandroupolis, Greece

**Keywords:** pharmacogenomics, precision medicine, fluoropyrimidines, 5-fluorouracil, capecitabine, tegafur, toxicity, polygenic dosing algorithm

## Abstract

Fluoropyrimidines are chemotherapeutic agents widely used for the treatment of various solid tumors. Commonly prescribed FPs include 5-fluorouracil (5-FU) and its oral prodrugs capecitabine (CAP) and tegafur. Bioconversion of 5-FU prodrugs to 5-FU and subsequent metabolic activation of 5-FU are required for the formation of fluorodeoxyuridine triphosphate (FdUTP) and fluorouridine triphosphate, the active nucleotides through which 5-FU exerts its antimetabolite actions. A significant proportion of FP-treated patients develop severe or life-threatening, even fatal, toxicity. It is well known that FP-induced toxicity is governed by genetic factors, with dihydropyrimidine dehydrogenase (*DPYD*), the rate limiting enzyme in 5-FU catabolism, being currently the cornerstone of FP pharmacogenomics. *DPYD*-based dosing guidelines exist to guide FP chemotherapy suggesting significant dose reductions in *DPYD* defective patients. Accumulated evidence shows that additional variations in other genes implicated in FP pharmacokinetics and pharmacodynamics increase risk for FP toxicity, therefore taking into account more gene variations in FP dosing guidelines holds promise to improve FP pharmacotherapy. In this review we describe the current knowledge on pharmacogenomics of FP-related genes, beyond *DPYD*, focusing on FP toxicity risk and genetic effects on FP dose reductions. We propose that in the future, FP dosing guidelines may be expanded to include a broader ethnicity-based genetic panel as well as gene*gene and gender*gene interactions towards safer FP prescription.

## 1 Introduction

Fluoropyrimidines (FPs) are chemotherapeutic agents belonging to the antimetabolite drug class that are widely used in the treatment of solid tumors, including gastrointestinal (colorectal, liver, and pancreatic), head and neck, and breast cancer (Ioannou et al., 2021). FPs used in clinical practice are 5-fluorouracil (5-FU), and its prodrugs, capecitabine (CAP) and tegafur. FPs inhibit the biosynthetic process of DNA and RNA synthesis by directly incorporating into nucleic acids the active nucleotides fluorouridine triphosphate (FUTP) and fluorodeoxyuridine triphosphate (FdUTP). FPs can be administered as a monotherapy or in combination with other antineoplastic medications such as irinotecan, oxaliplatin, leucovorin, or biological therapies (Longley et al., 2003).

Both FP efficacy and toxicity rely on the final concentration of FUTP and FdUTP. FP efficacy as a first line or add-on therapy in different cancers is well-documented (García-Alfonso et al., 2021; Yagi et al., 2021; Kataria et al., 2022; Khankhel et al., 2022) and will not be further discussed herein. Toxicity, however, is a serious drawback of FP therapy. Approximately 10%–40% of FP-treated patients present with severe or life-threatening, even fatal, toxicity (Barin-Le Guellec et al., 2020; Deac et al., 2020); the most common reported toxicities are hematological and gastrointestinal (nausea, vomiting, diarrhea), whereas other complications of FP treatment include hand and foot syndrome (HFS) (Thorn et al., 2011). Death rates due to FP-induced toxicity are high; in France, approximately 150 patients die per year due to FP-toxicity (Barin-Le Guellec et al., 2020), whereas in the U.S.A approximately 1.300 FP-related deaths occur (Santos et al., 2017). The incidence and severity of FP-induced adverse drug reactions (ADRs) vary depending on multiple factors, including misadjusted dosing proportion and schedule, drug interactions and individual clinicopathological characteristics (Lam et al., 2016). Their effect on FP therapy is of utmost clinical importance since ADRs result in unavoidable dose reductions, delay of chemotherapeutic scheme administration or even chemotherapy termination and need for administration of alternative agents.

Pharmacogenomics is currently applied in guiding FP dosing aiming in reducing ADR incidence. Clinical implementation consists of *DPYD* genotyping encoding for dihydropyrimidine dehydrogenase (DPD), the rate limiting enzyme of 5-FU activation (Knikman et al., 2021). However, it appears that apart from *DPYD*, several polymorphisms exist in additional genes, mainly involved in FP metabolic pathway, that can affect FP response. In pharmacogenomics, it is well established that once a pharmacogene is clearly associated with drug dose, other genes may improve both dose prediction and sensitivity to ADRs, to a variable degree, after adjusting for the strongest association (Ragia et al., 2014). The polygenic dosing algorithm is being used for other drugs and vitamin K antagonists best fit within this approach (Verhoef et al., 2013; Ragia et al., 2017a; Ragia et al., 2017b). For this drug class the polygenic dosing algorithm includes genetic variations in *CYP2C9*/*VKORC1*, the principal enzymes that affect dose requirements, and, additionally, genetic information of secondary enzymes such as CYP4F2 and GGCX, that influence interindividual dose requirements after adjusting for *CYP2C9*/*VKORC1* (warfarindosing.org). In the present review we provide a comprehensive overview of the current data on FP pharmacogenomics, focusing on gene variants that increase risk for FP-induced toxicity and can potentially be used in FP dose decisions. We focus on genes beyond *DPYD* as we aim to identify additional genetic components and potential gene*gene and environment*gene interactions that contribute to FP response variability with the goal of assessing whether a polygenic FP dosing algorithm would improve targeted clinical use of the drug. The ultimate goal of this review is to propose the concept and the components of such an approach for individualized dose adjustments, in order to minimize FP-induced toxic events. The proposed components are presented in Table 1. It should be clarified, however, that the actual form and mathematical equation of such an algorithm is beyond the scope of the present work.

**TABLE 1:** Genetic variations that should be considered in any polygenic dosing clinical algorithm together with other non-genetic factors discriminating individuals at risk for FP-induced toxicity and in need for dose reduction or alternative therapy.

Gene	Affected FP	Variations	Risk for toxicity	Evidence, dose reduction recommendation	Other considerations
*DPYD*	5-FU, CAP, tegafur	*2A (rs3918290), *13 (rs55886062), c.2846T>A (rs67376798), c.1129–5923C>G (rs75017182, HapB3)	Increased overall toxicity, severe toxicity	Strong, available guidelines for dose reduction or alternative therapy	High positive predictive value, low sensitivity
		Additional deleterious and/or reduced activity alleles			Need for validation in different populations and identification of ethnicity-specific variants
					DPD phenotyping can also be used to guide FP dosing
*TYMS*	5-FU, CAP, tegafur	2R (rs45445694); 3′UTR 6bp ins/del (rs11280056)	Increased overall toxicity, severe toxicity	Strong, need for guidelines for dose reduction	Gene*gender interaction, priority in females
		Low expression alleles			
*ENOSF1*	5-FU, CAP, tegafur	rs2612091, additional LOF, low expression alleles	Increased overall toxicity, severe toxicity	Strong, need for guidelines for dose reduction	TYMS*ENOSF1 interaction
*CYP2A6*	tegafur	LOF, reduced activity alleles	Lack of response	Strong, need for clinical trials for dose modification (increase), need for guidelines for alternative therapy	Increased significance in Asian populations due to increased frequency of *CYP2A6* LOF alleles
*CES1*	CAP	Reduced activity alleles	HFS, toxicity	Moderate, potential dose reduction	Application in non-Asian populations
					The clinical significant variants need to be identified and validated in different populations
*CDA*	CAP	Reduced activity alleles	HFS, toxicity	Moderate, potential dose reduction	The clinical significant variants need to be identified and validated in different populations
*CES2*	CAP	Reduced activity alleles	HFS, toxicity	Low	The clinical significant variants need to be identified and validated in different populations
*DPYS*	5-FU, CAP, tegafur	Reduced activity alleles	Toxicity	Low	Analysis in cases of extreme toxicity in absence of other gene polymorphisms
*UPB1*	5-FU, CAP, tegafur	Reduced activity alleles	Toxicity	Low	Analysis in cases of extreme toxicity in absence of other gene polymorphisms
*MTHFR*	5-FU, CAP, tegafur	LOF, reduced activity alleles	Toxicity	Extremely low	Gene*gender interaction, potential association in females
*TYMP*	CAP	Reduced activity alleles	HFS, toxicity	Extremely low	
*UMPS*	5-FU, CAP, tegafur	Reduced activity alleles	HFS, toxicity	Extremely low	

FPs, fluoropyrimidines; *DPYD*, dihydropyrimidine dehydrogenase; 5-FU, 5-fluorouracil; CAP, capecitabine; LOF, loss-of-function; *ΤΥΜS*, thymidylate synthase; *ENOSF1*, enolase superfamily member 1; *CYP2A6*, cytochrome P450 isoenzyme 2A6; *CES*, carboxylesterases; HFS, hand and foot syndrome; *CDA*, cytidine deaminase; *DPYS*, dihydropyrimidinase; *UPB1*, β-ureidopropionase 1; *MTHFR*, methylene tetrahydrofolate reductase; *TYMP*, thymidine phosphorylase; *UMPS*, uridine monophosphate synthetase.

## 2 Fluoropyrimidine pharmacokinetic and pharmacodynamic pathways

**FIGURE 1:**
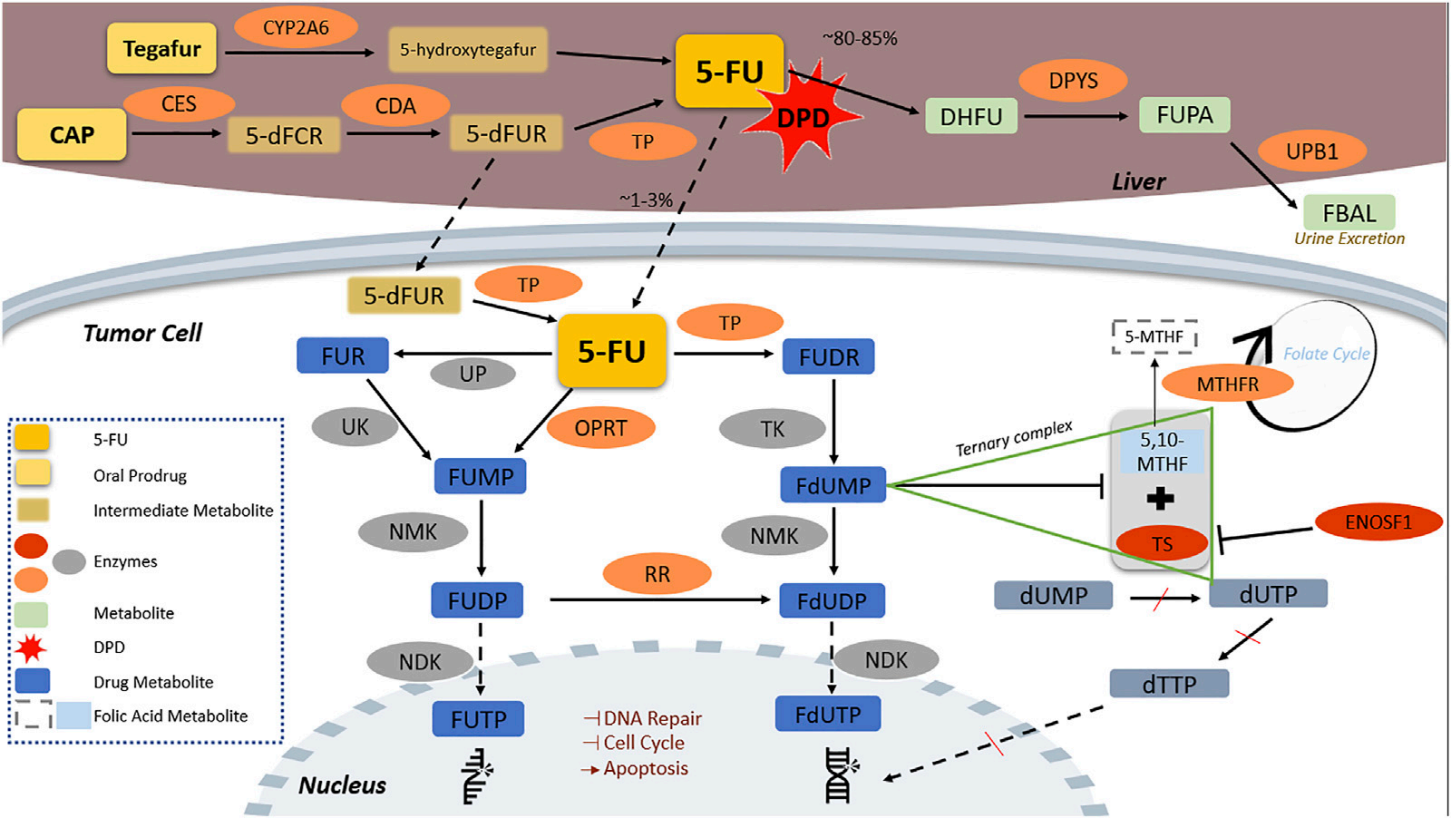
Fluoropyrimidine pharmacokinetic and pharmacodynamic pathway. CAP is transformed by CES to 5-dFCR which turns into 5-dFUR by CDA and then to its active form of 5-FU by TP. Tegafur, is metabolized in the liver by CYP2A6 to the unstable form of 5-hydroxytegafur, which spontaneously turns into 5-FU. 5-FU is catabolized by DPD to inactive metabolites in the liver; DPYS catabolizes DHFU to FUPA and UPB1 turns FUPA to FBAL which is excreted in the urine. The remaining 5-FU (approximately 1%–3% of initially administered) ends to the fluorine-substituted derivatives of uracil, FUTP and FdUTP, formed through two distinct pathways in which several enzymes are implicated. FUTP and FdUTP incorporate into RNA and DNA sequence inhibiting thus nucleic acid synthesis. FdUTP additionally inhibits the ternary complex of MTHFR substrate 5,10-MTHF with TS, which converts dUMP to dUTP and dTTP, a vital precursor for DNA replication and repair, inducing thereby cell apoptosis. ENOSF1 downregulates TS expression. 5,10-MTHF, 5,10-methylene tetrahydrofolate; 5-dFCR, 5-deoxy-5-fluorocytidine; 5-dFUR, 5-deoxy-5-fluorouridine; 5-FU, 5-fluorouracil; 5-MTHF, 5-methyltetrahydrofolate; CAP, capecitabine; CDA, cytidine deaminase; CES2, carboxylesterase 2; CYP2A6, cytochrome P450 2A6; DHFU, 5-fluoro-5,6-dihydrouracil; DPD, dihydropyrimidine dehydrogenase; DPYS, dihydropyrimidinase; dTTP, deoxythymidine triphosphate; dUMP, deoxyuridine monophosphate; dUTP, deoxyuridine triphosphate; ENOSF1, enolase superfamily member 1; FBAL, α-fluoro-β-alanine; FdUDP, fluorodeoxyuridine diphosphate; FdUMP, fluorodeoxyuridine monophosphate; FdUTP, fluorodeoxyuridine triphosphate; FUDP, fluorouridine diphosphate; FUDR, fluorodeoxyuridine; FUMP, fluorouridine monophosphate; FUPA, fluoro-β-ureidopropionate; FUR, fluorouridine; FUTP, fluorouridine triphosphate; MTHFR, methylenetetrahydrofolate reductase; NDK, nucleotide diphosphate kinase; NMK, nucleotide monophosphate kinase; OPRT, orotate phosphoribosyltransferase; RR, ribonucleotide reductase; TK, thymidine kinase; TP, thymidine phosphorylase; TS, thymidylate synthase; UK, uridine kinase; UP, uridine phosphorylase; UPB1, β-ureidopropionase 1.

Pharmacokinetics and pharmacodynamics of the three FPs clinically used, 5-FU, CAP, and tegafur, are complex but well characterized and have been described elsewhere in detail (Thorn et al., 2011). We briefly present herein the enzymes that are involved in each step, from drug activation to drug action, since these enzymes are further discussed as for their potential pharmacogenomic importance. FP pharmacokinetic and pharmacodynamic pathways and the key enzymes involved are presented in Figure 1.

In brief, CAP passes through the intestinal tract unchanged and is transformed to 5-deoxy-5-fluorocytidine (5-dFCR) by liver carboxylesterases (CES). 5-dFCR turns into 5-deoxyfluorouridine (5-dFUR) by cytidine deaminase (CDA) and then to its active form of 5-FU by thymidine-phosphorylase (TP, encoded by *TYMP*). The latter reaction is also catalyzed in cancer tissues (Lam et al., 2016). The second prodrug, tegafur, is metabolized in the liver by CYP2A6 to the unstable form of 5-hydroxytegafur, which spontaneously turns into 5-FU (Deac et al., 2020).

Approximately 80%–85% of the administered 5-FU is catabolized to inactive metabolites in the liver with DPD holding the key role in this critical step. 5-FU is converted to 5,6-dihydro-5-fluorouracil (DHFU) by DPD; DHFU is catabolized to fluoro-β-ureidopropionate (FUPA) and finally to α-fluoro-β-alanine (FBAL), which is excreted in the urine within 24 h (Miura et al., 2010). The enzymes involved in this process are dihydropyrimidinase (DPYS) and β-ureidopropionase 1 (UPB1). A proportion as low as 1%–3% of the administered 5-FU ends to fluorine-substituted derivatives of uracil fluorouridine triphosphate (FUTP) and fluorodeoxyuridine triphosphate (FdUTP) that mediate the therapeutic and cytotoxic effects of the drug. FUTP and FdUTP are formed through two distinct pathways (Thorn et al., 2011). Several enzymes, such as uridine kinase (UK), nucleotide monophosphate kinase (NMK), nucleotide diphosphate kinase (NDK), orotate phosphoribosyltransferase (OPRT), ribonucleotide reductase (RR), thymidine kinase (TK), and uridine phosphorylase (UP) are involved in these steps (Figure 1).

FUTP and FdUTP incorporate into RNA and DNA sequence thus inhibiting nucleic acid synthesis (Longley et al., 2003). FdUTP also inhibits thymidylate synthase (TS), the enzyme that binds deoxyuridine monophosphate (dUMP) (a precursor of dUTP) and this inhibition results in dUMP accumulation, deoxythymidine monophosphate (dTMP) suppression, and, subsequently, depletion of deoxythymidine triphosphate (dTTP), a vital precursor for DNA replication and repair. Reduced dTTPs disrupt DNA synthesis and repair, inducing thereby cell apoptosis (Lam et al., 2016; Ioannou et al., 2021). Importantly, additional enzymes, such as enolase superfamily member 1 (ENOSF1) and methylenetetrahydrofolate reductase (MTHFR) interfere with FP action, through interactions with TS and intermediate metabolites (Lam et al., 2016; De Mattia et al., 2019b). Finally, enzymes involved in DNA repair, cell cycle, and apoptosis mediate the cellular response to FPs (De Angelis et al., 2006).

As it appears, multiple enzymes are involved in FP pharmacokinetic and pharmacodynamic pathways. Identifying genetic variations affecting the activity of these enzymes and interfering with the final concentration of FUTP and FdUTP, holds promise to improve FP pharmacogenomics. Assumptions are based on published data presented in detail in this review.

## 3 Fluoropyrimidine pharmacogenomics

### 3.1 *DPYD* in the prime time

DPD is the primary enzyme for 5-FU breakdown, whereas DPD deficiency is known to be a leading cause of severe FP-induced toxicity (White et al., 2022). Genetic variations in the *DPYD* gene can severely affect DPD activity and are the cornerstone of FP pharmacogenomics. Over 160 polymorphisms have been identified in *DPYD*. However, four *DPYD* variants, namely, DPYD*2A (rs3918290), DPYD*13 (rs55886062), c.2846T>A (rs67376798), and c.1129–5923C>G (rs75017182, HapB3) currently drive FP dosing strategy. The association of these variations in *DPYD* both with FP-induced severe toxicity and the need for dose reductions is firmly established. Results of a recent meta-analysis showed that carriers of pathogenic *DPYD* gene variants have a severely increased (over 25-fold) risk of treatment-related death and a prevalence of 3.7% of treatment-related mortality (Sharma et al., 2021). Advances in clinical *DPYD* genotyping include guidelines for *DPYD* genotype and FP dosing released by Clinical Pharmacogenetics Implementation Consortium (CPIC) (Amstutz et al., 2018) and the Dutch Pharmacogenetics Working Group (DPWG) (Lunenburg et al., 2020). Both CPIC and DPWG recommend reductions in *DPYD* defective patients ranging from 25% to 50% based on *DPYD* allele combination (*DPYD* activity score) and, additionally, avoidance of FP use in patients with a complete lack of DPD activity (activity score 0) (Amstutz et al., 2018; Lunenburg et al., 2020).

Overall, the benefits and cost-effectiveness of implementing *DPYD* genotyping prior to FP dosing have been shown in several prospective trials and meta-analyses (Meulendijks et al., 2015; Henricks et al., 2018; Brooks et al., 2022; Glewis et al., 2022; van der Wouden et al., 2022). Regulatory agencies such as the Food and Drug Administration (FDA) and European Medicine Agency (EMA), have moved towards recognizing the importance of the application of *DPYD* genotyping in clinical practice. FDA has included drug label warnings describing the association of DPD deficiency with FP toxicity and the need for potential dose adjustments, however, no further details are provided (FDA, 2015a; FDA, 2016). EMA, since April 2020, has recommended that patients should be tested for the lack of DPD enzyme before starting FP treatment, either by measuring the level of uracil in the blood (phenotyping) or by genotyping for DPYD*2A, *13, c.2846T>A, and c.1129–5923C>G alleles (EMA, 2020). DPD phenotyping is a strategy that can be used to identify patients at risk for FP-induced toxicity (Deac et al., 2020), albeit has yet to be broadly incorporated in routine clinical practice. However, when phenotypic data is available, this information can guide FP-dosing decisions (Table 1). Following EMA recommendation, upfront *DPYD* genotyping has been endorsed by several countries in Europe, including Spain, Switzerland, Germany, Austria, the United Kingdom, and the Netherlands (Martens et al., 2019; Wörmann et al., 2020; Begré et al., 2022; Etienne-Grimaldi et al., 2022; García-Alfonso et al., 2022; Wang et al., 2022; White et al., 2022).

Despite the undisputable significance of *DPYD* variations on FP dosing decisions, *DPYD* polymorphisms can only explain a small percentage of FP-induced ADRs. The combined sensitivity of the major *DPYD* variants to predict grade 3–4 5-FU related toxicity is relatively low; results from *DPYD* genotyping in more than 2,500 5-FU treated patients (33.0% > 3 grade 5-FU toxicity) showed that DPYD*2A, D949V, and I560S variants resulted in 5.3% sensitivity, 99.4% specificity, 81.8% positive predictive value and 68.0% negative predictive value for grade ≥3 5-FU AE prediction (Lee et al., 2014). Considering the scarcity of the four recommended for genotyping *DPYD* variants [lower than 5% in European populations (Innocenti et al., 2020)] a missing heritability component exists within *DPYD* gene. Thus, the proportion of FP-treated patients that experience any grade of FP-induced toxicity is higher than the percentage of *DPYD* variant allele carriers treated with FPs, meaning that a negative *DPYD* test cannot exclude the possibility of experiencing drug-induced ADRs.

To improve sensitivity, *DPYD* gene is currently under intense study for the identification of additional, population-specific, clinically relevant variants, rare mutations, and/or copy number variations (Palles et al., 2021; De Luca et al., 2022; De Mattia et al., 2022; Kanai et al., 2022; Wigle et al., 2023). Indeed, results from a large scale genetic analysis in patients from the QUASAR2 clinical trial revealed three, additional to the CPIC-recommended, variants, namely, rs12132152, rs12022243, and p.Ala551Thr, that were associated with CAP-toxicity (Rosmarin et al., 2015). The combination of additional deleterious, albeit rare, *DPYD* variants can increase sensitivity (Etienne-Grimaldi et al., 2017), however, still, the estimated 10%–15% of DPD-linked FP-related AEs cannot be uniquely explained by *DPYD* low-frequency variants.

### 3.2 Beyond *DPYD*: most prominent associations

#### 3.2.1 Thymidylate synthase, *ΤYΜS*

Thymidylate synthase (TS), encoded by *TYMS* gene, influences abundance of dTTPs while it is also inhibited by FdUMP (Figure 1). *TYMS*, therefore, is both necessary for DNA synthesis and a target of FPs (Hernando-Cubero et al., 2017). Patients overexpressing TS commonly present with 5-FU resistance, while patients with lower TS expression are better responders to the antitumor therapy being, however, also more vulnerable to drug-induced toxicity (Marsh, 2005). *TYMS* gene polymorphisms have been shown to alter TS expression and have been associated with FP treatment effectiveness and toxicity (Ioannou et al., 2021). Four common polymorphisms in *TYMS* gene located in *TYMS* untranslated regions (UTRs) are known to affect TS expression: *TYMS*-TSER 2R/3R (rs45445694), 2RG/2RC (rs183205964), 3RG/3RC (rs2853542) and a 6bp deletion allele (rs11280056) (Marsh, 2005; Lam et al., 2016; Xie et al., 2020).

Several *TYMS* polymorphisms have been extensively studied and are associated with FP-induced toxicity, suggesting that FP dose can be tailored to *TYMS* genotype. In the meta-analysis conducted by Rosmarin et al. (2014) *TYMS*-TSER 2R allele, 3′UTR 6bp ins/del variant and *TYMS* low activity genetic burden favor global CAP-induced toxicity (OR 1.36 for *TYMS*-TSER 2R allele, OR 1.25 for 3′UTR 6bp ins/del and OR 1.31 for *TYMS* genetic score). In a following meta-analysis in non-DPYD*2A carriers, low *TYMS* expression genotypes (3RC/2RC, 2RG/2RC and 2RC/2RC) were associated with global severe toxicity (OR 3.0) and toxicity-related hospitalization (OR 3.8) (Meulendijks et al., 2016). Recently, in the largest, so far, meta-analysis including more than 1,900 patients, *TYMS* 2R allele was associated with grade 3 HFS (OR 1.50), while *TYMS* 3′UTR 6bp ins/del was associated with overall toxicity (OR 1.21) and grade 3 HFS (OR 1.41) (Hamzic et al., 2020). Schaerer et al. (2020) have proposed that *TYMS* polymorphisms affect the number of upstream stimulatory factor (USF1) binding sites in the gene. The authors have shown that patients with fewer USF1-binding sites have increased risk for early-onset gastrointestinal toxicity (OR 1.66) and severe gastrointestinal toxicity (OR 1.74 after adjustment for *DPYD*) in response to 5-FU treatment.

*TYMS* is considered a gene of importance in FP pharmacogenomics, however, no predictive strategies have yet been clinically applied (Amstutz et al., 2018). It appears, therefore, that *TYMS* variants that reduce TS expression can improve the pharmacogenomic *DPYD*-guided dosing strategy and it is anticipated that soon *TYMS* will be the second FP-dosing associated pharmacogene.

#### 3.2.2 Enolase superfamily member 1, *ENOSF1*

*ENOSF1* gene encodes for the mitochondrial enolase superfamily member 1, an enzyme that catalyzes the conversion of L-fuconate to 2-keto-3-deoxy-L-fuconate. It was initially identified as a gene coding for an antisense RNA that downregulates TS expression via promoting *TYMS* mRNA degradation (Dolnick, 1993; Dolnick et al., 1996; Chu and Dolnick, 2002). *ENOSF1* and *TYMS*, both located on chromosome 18, show a partial overlap in their sequences and are transcribed in opposite directions. Several polymorphisms have been identified within *ENOSF1* and have been studied as for their functional effect on *TYMS* gene expression and consequently on FP-induced toxicity.

The seminal report on the association of *ENOSF1* and CAP toxicity was provided by Rosmarin et al. (2015). The authors have sequenced 25 CAP/5-FU pathway genes in a cohort of 968 patients, participants of QUASAR2 study. They have found a significant association of *ENOSF1* rs2612091A intronic variant, lying 10 kb downstream of *TYMS*, with CAP toxicity (OR = 1.59) (Rosmarin et al., 2015). The authors additionally proposed that *ENOSF1* rs2612091 polymorphism affects TS protein activity rather than *TYMS* expression (Rosmarin et al., 2015). The association of *ENOSF1* rs2612091 polymorphism with FP-induced toxicity was later replicated by a study in a cohort of 239 CAP-treated patients showing that *ENOSF1* rs2612091 was associated with HFS (OR = 2.28) (García-González et al., 2015). Further studies have shown that *ENOSF1* rs2612091 is associated with shorter overall survival (Meulendijks et al., 2017) and treatment non-response (Arjmandi et al., 2022), whereas *ENOSF1*/*TYMS* rs699517 polymorphism was associated with CAP-induced severe nausea/vomiting, anorexia and fatigue (Pellicer et al., 2017). A meta-analysis of *ENOSF1* rs2612091 association with FP-induced toxicity has demonstrated that *ENOSF1* rs2612091 was associated with severe HFS (OR = 1.64) independently of *TYMS* variants (Hamzic et al., 2020).

*ENOSF1* appears as a promising marker for prediction of FP-induced toxicity. While we expect that more studies will be published on *ENOSF1* and its association with dose requirements, available associations show a high degree of consistency that favors incorporation of *ENOSF1* pharmacogenomics in clinical practice. It is noteworthy that the *ENOSF1* rs2612091 effect appears independently of the effect of *TYMS* polymorphisms. More importantly, Palles et al. (2021) have shown that when *ENOSF1* rs2612091 is integrated to the *DPYD*-based prediction model for FP-induced toxicity, it significantly improves prediction of global toxicity, hematological toxicity, HFS and diarrhea. It appears that *ENOSF1* may have a place in any future polygenic algorithm for FP-dosing.

#### 3.2.3 Methylene tetrahydrofolate reductase, *MTHFR*

*MTHFR* is also an extensively-studied pharmacogene in terms of its effects on FP treatment response. MTHFR irreversibly catalyzes 5,10 MTHF conversion to 5-methyltetrahydrofolate (5-MTHF), the primary methyl donor responsible for DNA methylation. As in the case of TS, FdUMP forms a ternary complex with MTHFR substrate, 5,10-MTHF. Therefore, decreased MTHFR activity leads to increased 5,10-MTHF concentration and can enhance TS inhibition and FP activity, increasing this way the risk for FP-induced ADRs (Lam et al., 2016; De Mattia et al., 2019b).

Two *MTHFR* polymorphisms, which are in linkage disequilibrium, −677C>T (rs1801133) and −1298C>A (rs1801131), lead to impaired MTHFR enzymatic activity and have been extensively studied in association with FP-induced toxicity (Ramalakshmi et al., 2016). A large number of studies focused on *MTHFR* pharmacogenomics have been published, however, to date, the impact of *MTHFR* variations on FP-induced toxicity is still not clear (Toffoli and De Mattia, 2008; De Mattia and Toffoli, 2009). Results of meta-analyses, as reviewed by Campbell et al. (2016), show isolated associations within studies rather than a universal effect of *MTHFR* on FP-induced toxicity. In a more recent meta-analysis, Zhong et al. (2018) also conclude that *MTHFR* polymorphisms could not be considered as reliable factors for predicting FP clinical response. A potential limitation in *MTHFR* case may rely on the folate cycle, a pathway involving several enzymes that can modulate MTHFR potency; folylpolyglutamate synthetase enzyme (FPGS) and gamma-glutamyl hydrolase (GGH) are such enzymes interfering with intracellular folate concentrations. Studies on the association of *FPGS* and *GGH* variations on chemotherapy response have started to emerge (Kim et al., 2008; Fernandes et al., 2021) and we anticipate that more information will be gathered in the near future.

Based on these findings, more research is required for the integration of *MTHFR* genetic information in a polygenic FP-dosing algorithm. Future research, however, should focus on limitations that may hinder the effect of *MTHFR* on FP response, such as heterogeneity of cancer patients recruited in different studies, variable 5-FU based regimens and interactions of gene with nutrition, ethnicity and other environmental factors.

### 3.3 Additional genetic variations towards a polygenic dosing algorithm

For FPs, beyond *DPYD* and the abovementioned extensively studied genes, several polymorphisms in other genes have been assessed as for their potential association with FP-induced toxicity. In the following sections, results of studies assessing the pharmacogenomic implication of genes participating in FP response pathways are described and critically reviewed. Genes that show a high degree of consistency in their association with FP response across different studies are summarized in Table 1.

#### 3.3.1 Carboxylesterases, CES

Carboxylesterases (CES) belong to the serine enzyme superfamily and they metabolize and activate several drugs, including CAP. In humans, five CES enzymes are coded (CES1-CES5); among them, CES1 and CES2 have a role in CAP metabolism, catalyzing 5-DFCR formation with similar catalytic efficiency (Ramalakshmi et al., 2016). Both *CES2* and *CES1* polymorphisms have been found to influence CAP treatment outcomes. Results of relevant studies are described in Table 2 and Table 3, respectively.

**TABLE 2:** Studies examining the effect of *CES2* gene polymorphisms on CAP-induced toxicity.

Examined *CES2* variants	Study population	Endpoints	Results	Ref
−823 C>G; −854 G>C; 5841 G>A; 6046 G>A; 6174 G>A; 6320 G>A	136 CAP-treated metastatic breast or colorectal cancer patients, 39% grade 3–4 toxicity (n = 123 genotyped for *CES2*)	Grade 3–4 toxicity	A trend towards association between 6046 G>A and diarrhea incidence (*p* = 0.09)	Ribelles et al. (2008)
rs11075646 (−823 C>G); rs2241409; rs11568314; rs11568311	111 CAP-treated HER2/neu- negative metastatic breast cancer patients	Grade 3–4 toxicity	−823 C>G associated with grade 3–4 HFS (OR = 4.49, 95% CI 1.43–14.14, *p* = 0.01)	Martin et al. (2015)
rs11075646; rs2241409; rs11568314; rs11568311	QUASAR2 trial (ISRCTN45133151)	Grade 0–2 vs. grade 3+ toxicity	No association found at the threshold set in the study	Rosmarin et al. (2014)
	927 post-operative stage II/III colorectal cancer patients administered CAP in monotherapy or in combination with bevacizumab			
Meta-analysis: rs11075646; rs11568314; rs2241409; rs11568311; 6046 G>A; 6320 G>A	Rs11075646 genotyped in 881 patients, while rs2241409 rs11568311			
	genotyped in 442 and rs11568314			
	in 439 patients			
rs11075646; rs2241409; rs11568314; rs11568311	130 breast and colorectal cancer patients	Grade 3 HFS	No association found	Caronia et al. (2011)
rs11075646	188 women with HER2-negative metastatic breast cancer randomized on CAP-including or CAP-excluding schemes, n = 184 genotyped for rs11075646	HFS, first dose reduction	*CES2* rs11075646 WT genotype benefit from CAP-including scheme (progression-free survival benefit)	Lam et al. (2018)
			No association with HFS or first dose reduction	
rs11075646	26 metastatic breast adenocarcinoma female patients	Grade 1–3 toxicity	No association found	Rudek et al. (2013)
rs11075646	446 patients treated with CAP monotherapy or in combination with oxaliplatin	HFS occurrence	No association found	de With et al. (2023)

*CES2*, carboxylesterase 2; CAP, capecitabine; HER2, human epidermal growth factor receptor 2; HFS, hand and foot syndrome; OR, odds ratio; WT, wild-type.

**TABLE 3:** Studies examining the effect of *CES1* gene polymorphisms on CAP-induced toxicity.

Examined *CES1* variants	Study population	Endpoints	Results	Ref
rs2244613; rs2244614; rs3217164; rs7187684; rs1186118; rs71647871	144 cancer patients, CAP monotherapy or in combination regimens	Grade 0–1 vs. 2 vs. 3–4 adverse events (HFS, diarrhea), overall toxicity	Associated with overall toxicity: 1165–41 C>T (*p* = 0.001), 690 + 129del (*p* < 0.001), rs1186118 and rs7187684 (*p* = 0.012 each), 1165–33 C>A (*p* = 0.013)	Hamzic et al. (2017)
			A3 minor-alleles-haplotype associated with overall toxicity in additive (OR 2.18, 95% CI 1.19–4.00, *p* = 0.012) and recessive genetic model (OR 10.25, 95% CI 2.12–49.43, *p* = 0.0038)	
			A1 major alleles haplotype was protective against CAP-induced toxicity (OR 0.60, 95% CI 0.36–0.99, *p* = 0.047)	
rs2244613; rs2244614; rs3217164	446 patients treated with CAP monotherapy or in combination with oxaliplatin	HFS occurrence	1165–33 C>A variation associated with HFS ≥ grade 2 (OR 1.888; 95% CI 1.075–3.315, *p* = 0.027)	de With et al. (2023)
rs7498748	301 colorectal cancer patients, CAP monotherapy or in combination regimens	Grade ≥ 2 adverse events, overall toxicity, treatment administration delay	No association found	Pellicer et al. (2017)
rs2244613; rs2244614; rs3217164; rs7187684; rs1186118	36 Japanese postoperative or metastatic colorectal cancer patients	Overall toxicity (grade ≥ 3)	No association found	Matsumoto et al. (2020)
rs2244613; rs2244614; rs3217164; rs7187684; rs1186118	338 Chinese colorectal and gastric cancer administered FPs	Hematological, hepatic, gastrointestinal toxicity, and HFS	No association found	Liu et al. (2021)

*CES1*, carboxylesterase 1; CAP, capecitabine; HFS, hand and foot syndrome; OR, odds ratio; FPs, fluoropyrimidines.

##### 3.3.1.1 CES2

Historically, *CES2* polymorphisms were studied prior to *CES1* polymorphisms for their potential association with CAP response in terms of toxicity incidence. Ribelles et al. (2008) have analysed *CES2* −823 C>G, −854 G>C, 5841 G>A, 6046 G>A, 6174 G>A, and 6320 G>A polymorphisms in relation to CAP response and severe toxicity in a prospective study in 123 CAP-treated patients. *CES2* 6046 G>A showed a non-significant trend (*p* = 0.09) towards increased incidence of grade 3–4 diarrhea. Martin et al. (2015) have assessed the association of *CES2* rs2241409, rs11568314, rs11568311, and rs11075646 (−823 C>G) polymorphisms with grade 3–4 toxicity in 111 women with advanced breast cancer treated with CAP monotherapy. It was found that rs11075646 (5′UTR 823 C>G) minor allele was associated with increased risk for grade 3–4 HFS (OR 4.49, *p* = 0.01). Evidence for association of *CES2* polymorphisms with CAP-induced HFS also derive from QUASAR2 clinical trial, a study on 927 CAP-treated patients. Rs2241409 showed a trend towards association with grade ≥3 HFS (*p* = 0.035), however, this association is not statistically significant at the threshold set in the study (Rosmarin et al., 2014). There appears to be a tendency towards a weak association of *CES2* polymorphisms with HFS, however, these results were not replicated in several other studies (Caronia et al., 2011; Rudek et al., 2013; Lam et al., 2018; de With et al., 2023).

Overall, it appears that results accumulated so far do not support a role of *CES2* polymorphisms on FP-induced toxicity. The potential association of *CES2* variations with HFS merits further study.

##### 3.3.1.2 CES1

*CES1* variants have also been studied in relation to CAP-induced toxicity (Table 3). Hamzic et al. (2017) examined the potential role of CAP-activating genes (*CES1*, *CES2*, *TYMP*, *UPP1,* and *UPP2*) in severe (grade 3–4) early-onset toxicity in 144 cancer patients administered CAP. Patients were genotyped for six *CES1* polymorphisms; rs2244613, rs2244614, rs3217164, rs7187684, rs1186118, and rs71647871. With the exception of rs71647871, all studied *CES1* polymorphisms were significantly associated with grade 2–4 CAP-induced toxicity. In multivariate analysis (adjusted for *DPYD* risk variants) the haplotype encompassing rs2244613, rs2244614, rs3217164, rs7187684, and rs1186118 minor alleles was an independent predictor of CAP-induced toxicity. More recently, *CES1* 1165–33 C>A variation was strongly associated with HFS ≥ grade 2 (OR 1.888, *p* = 0.027) in a study including 446 patients of whom 32.7% developed HFS (17.3%≥ grade 2) (de With et al., 2023). It should be noted that studies conducted in Asian populations did not find an association of various *CES1* polymorphisms and CAP-induced toxicity (Matsumoto et al., 2020; Liu et al., 2021).

Whether the association of *CES1* with FP-response is limited in non-Asian populations needs to be further investigated. Following the example of *DPYD* for which it has been reported that the registered CPIC variations have only a minor role in FP-related toxicity in an Asian population (Kanai et al., 2022), we propose that *CES1* could be initially incorporated in a polygenic FP dosing algorithm for other ancestries until firm conclusions are drawn on its potential effect on FP response in Asian populations. It appears that multiple *CES1* variants should be considered as predictive factors to CAP-induced HFS. The exact *CES1* polymorphism combination as well as the potential interaction of CES1*CES2 genes need to be verified.

#### 3.3.2 Cytidine deaminase, *CDA*

Cytidine deaminase (CDA) has a crucial role in pharmacological activation of CAP to 5-FU and exhibits a highly variable enzymatic activity among individuals. This variation can be partially attributed to its different haplotypes which can lead to an increase or decrease of 5-FU concentration (Morita et al., 2003). The results of studies examining the association of *CDA* variants with CAP-induced toxicity are summarized in Table 4.

**TABLE 4:** Studies examining the effect of *CDA* gene polymorphisms on CAP-induced toxicity.

Examined *CDA* variants	Study population	Endpoints	Results	Ref
−943del/insC; 1052 A>C; 575 C>T; 771 C>G; 794 G>A; 942 C>G	136 CAP-treated metastatic breast or colorectal cancer patients, 39% grade 3–4 toxicity (*n* = 123 genotyped for *CDA*)	Grade 3–4 toxicity	A trend towards association between −943insC allele and grade 3 HFS (21% of the carriers of one or two insertion alleles vs. 8% for the wild type patients, *p* = 0.07)	Ribelles et al. (2008)
rs532545 (−451 C>T); rs602950 (−92 A>G); rs2072671; rs3215400 (−943del/insC); rs603412	130 CAP-treated breast and colorectal cancer patients	Grade 3 HFS	−451T associated with HFS (OR 2.02, 95% CI 1.02–3.99, *p* = 0.039) in additive genetic model	Caronia et al. (2011)
			−943insC associated with lower risk for HFS in additive (OR 0.51, 95% CI 0.27–0.95, *p* = 0.028) and recessive genetic model (OR 0.37, 95% CI 0.16–0.86, *p* = 0.020)	



−451C>T; −943del/insC; −92 A>G; 79 A>C	244 patients with gastrointestinal cancer administered CAP regimens	Adverse events, overall toxicity	−451T associated with diarrhea grade 2–4 (OR 2.3, 95% CI 1.3–4.2, *p* = 0.0082)	Loganayagam et al. (2013)
			−92G associated with grade 2–4 diarrhea (*p* = 0.002) and dehydration (*p* = 0.042)	


rs2072671 (79 A>C)	239 colorectal cancer patients administered CAP in monotherapy (*n* = 69) or in combination regimens (*n* = 170)	Grade ≥ 3 toxicities (HFS, diarrhea, hematological toxicity), overall toxicity, treatment reduction/delay/withdrawal	79A associated with overall toxicity in univariate (*p* = 0.008) and in multivariate analysis (OR 1.84, 95% CI 1.06–3.18, *p* = 0.029)	García-González et al. (2015)
−182 G>A; 79 A>C; 435 C>T	301 colorectal cancer patients administered CAP in monotherapy or in combination regimens	Grade ≥ 2 adverse events, overall toxicity, and treatment administration delay	79AA associated with risk of toxicity (OR 1.89) and HFS (OR 3.83)	Pellicer et al. (2017)
			435T associated with higher bilirubin (OR 8.621, 95% CI 1.058–70.247, *p* = 0.044)	
			−182A associated with treatment administration delay (OR 2.743, 95% CI 1.346–5.588, *p* = 0.005)	
1172 G>A; −451 C>T; −92 A>G; 435 C>T; 265 A>T; 266+242 A>G; 79 A>C	144 cancer patients administered CAP in monotherapy or in combination regimens	Grade 0–1 vs. 2 vs. 3–4 toxicities (HFS, diarrhea), overall toxicity	−92G, −451T associated with grade 2–4 diarrhea (OR 4.40, 95% CI 1.34–14.5, *p* = 0.015, and OR 4.29, 95% CI 1.30–14.2 *p* = 0.017, respectively)	Hamzic et al. (2017)
			1172A associated with HFS (OR 3.50, 95% CI 1.13–10.9, *p* = 0.030)	
			266 + 242A>G associated with overall toxicity (OR 2.00, 95% CI 1.00–3.96, *p* = 0.048) and diarrhea (OR 3.33, 95% CI 1.26–8.81, *p* = 0.015)	
			−451T, −92G, 33delC and 79C haplotype associated with diarrhea (OR 2.09, 95% CI 1.07–4.10, *p* = 0.032)	



−451 A>G; −92 C>T; 79 A>C; 1172 G>A	322 colorectal or gastric cancer Chinese patients administered CAP in monotherapy or in combination regimens	Toxicity	−451AA genotype associated with less hepatotoxicity (OR 0.200, 95% CI 0.045–0.895, *p* = 0.035) and grade 3–4 hematological toxicity (OR 0.205, 95% CI 0.045–0.927, *p* = 0.039)	Liu et al. (2019)



rs2072671; rs603412; rs10916825	446 patients treated with CAP monotherapy or in combination with oxaliplatin	HFS occurrence	266 + 242 A>G (rs10916825) was associated with HFS ≥ grade 2 (OR 1.865, 95% CI 1.087–3.200, *p* = 0.024)	de With et al. (2023)


−451 C>T; −92 A>G; 79 A>C; −943del/insC; rs603412	111 CAP treated HER2/neu- negative metastatic breast cancer patients, (50 control patients +61 experimental patients)	Grade 3–4 toxicity	No association found	Martin et al. (2015)


−451 C>T; −943del/insC	188 women with HER2-negative metastatic breast cancer randomized on CAP-including (*n* = 93) or CAP-excluding schemes (*n* = 95)	HFS	No association found	Lam et al. (2018)

−451 C>T; 79 A>C; −205 C>G	QUASAR2 trial (ISRCTN45133151)	Grade 0–2 vs. grade ≥3 adverse events	No association found at the threshold set in the study	Rosmarin et al. (2014)
	927 post-operative stage II/III colorectal cancer patients administered CAP in monotherapy or in combination regimens			
	−451C>T and 79A>C genotyped in 927 patients, −205C>G			
Meta analysis: 79 A>C; -943del/insC; 575 C>T; 771 C>G; −205 C>G; −92 A>G; 794 G>A	genotyped in 89 patients			

*CDA*, cytidine deaminase; CAP, capecitabine; HFS, hand and foot syndrome; OR, odds ratio; ADRs, adverse drug reactions; HER2, human epidermal growth factor receptor 2; 5-FU, 5-fluoruracil.

Ribelles et al. (2008) were the first to report a trend towards increased risk for grade 3 HFS in *CDA* −943insC allele carriers (*p* = 0.07); study cohort consisted of 123 patients with metastatic breast or colorectal cancer. Caronia et al. (2011) genotyped *CDA* −451C>T, −92A>G, 79A>C, −943delC, and −205C>A variants in 130 cancer patients and showed that −451T allele predicted severe HFS (OR 2.02, *p* = 0.039). Interestingly, −943insC allele was found to have a protective effect against HFS (OR 0.51, *p* = 0.028). Those results were further verified through *CDA* expression analysis in 89 lymphoblastoid cell lines from Caucasian healthy individuals, in which −943delC homozygous cell lines had >3-fold increased CDA mRNA expression (compared to the carriers of the insertion allele). In a larger retrospective study enrolling 430 patients with gastrointestinal cancer, Loganayagam et al. (2013) genotyped *CDA* −451C>T, −943delC, −92A>G, and 79A>C polymorphisms in a subcohort of 244 patients receiving CAP-based chemotherapy, who did not carry *DPYD* defective variants. In regression analysis, −92G allele was associated with grade 2–4 diarrhea (*p* = 0.002) and dehydration (*p* = 0.042), whereas −451T was associated with grade 2–4 diarrhea (*p* = 0.0082).

The association of *CDA* 79A>C polymorphism with FP-induced toxicity was further studied by García-González et al. (2015) in a study including 239 CAP-treated patients. *CDA* 79A>C polymorphism was associated with overall toxicity (any toxicity grade ≥3) both in multivariate and univariate analysis (*p* = 0.029 and 0.008, respectively). Three *CDA* variants (−182G>A, 79A>C, and 435C>T) were further examined in another study, including 301 patients. *CDA* 435T allele was associated with hepatotoxicity (as estimated by higher bilirubin) (OR 8.62, *p* = 0.044), −182A with treatment administration delay due to ADRs (OR 2.743, *p* = 0.005) and 79A with both HFS (OR 3.83), and overall toxicity (OR 1.89) (Pellicer et al., 2017). *CDA* variants were also analyzed by Hamzic et al. (2017) in a cohort of 144 CAP-treated patients. After the coding and flanking areas of *CDA* were sequenced, seven variations (−1172G>A, −451C>T, −92A>G, 435C>T, 265A>T, 266 + 242A>G, and 79A>C) were further examined. The authors have shown that carriers of c.266 + 242A allele had increased risk for overall toxicity (OR 2.0, *p* = 0.048) and diarrhea (OR 3.33, *p* = 0.015). Additional associations with diarrhea were found for −451T, and −92G alleles (OR 4.29, *p* = 0.017 and OR 4.40, *p* = 0.015, respectively), and with the haplotype consisting of −451T, −92G, 1-33delC, and 79C alleles (OR 2.09, *p* = 0.032). Furthermore, −1172A was associated with HFS (OR 3.50, *p* = 0.030). The association of *CDA* -451A>G polymorphism with CAP-induced ADRs was also studied in 322 Chinese patients with gastrointestinal cancer. The authors have found that −451AA genotype has a rather protective effect on hematological (*p* = 0.039) and hepatological toxicity (*p* = 0.035) (Liu et al., 2019). More recently, in a study including 446 patients of whom 32.7% developed HFS (17.3% ≥ grade 2), *CDA* 266 + 242 A>G variation was strongly associated with HFS ≥ grade 2 (OR 1.865, *p* = 0.024) (de With et al., 2023).

Despite the extensive evidence of multiple *CDA* variants being associated with CAP-induced ADRs, these results were not replicated in all studies (Rosmarin et al., 2014; Martin et al., 2015; Lam et al., 2018). The low prevalence of studied variants may potentially explain this discordance in findings. For *CDA*, therefore, current evidence suggests that multiple gene variants exist that potentially affect CAP-induced ADRs and this gene should be considered for incorporation in a polygenic algorithm to adjust for CAP dose.

#### 3.3.3 Thymidine phosphorylase, *TYMP*

Thymidine phosphorylase (TP), encoded by *TYMP* gene, catalyzes the conversion of CAP to 5-FU and the subsequent conversion of 5-FU to FUDR in the metabolic pathway forming FdUMP (Bonotto et al., 2013). Apart from liver, this reaction takes also place in cancer cells (Figure 1); *TYMP* expression is higher in tumor cells compared to healthy tissue, leading to CAP preferential activation in tumor cells (Lam et al., 2016; Liu et al., 2021). The impact of *TYMP* polymorphisms on FP treatment response in terms of ADRs has been extensively investigated, however, the results appear contradictory (Table 5).

**TABLE 5:** Studies examining the effect of *TYMP* gene polymorphisms on CAP-induced adverse events.

Examined *TYMP* variants	Study population	Endpoints	Results	Ref
rs11479; rs112723255	253 colorectal cancer patients administered CAP (*n* = 159) or 5-FU (*n* = 94) in monotherapy or in combination regimens	Early dose modifications (delays or reductions), severe toxicity	rs11479 associated with early dose modifications (OR 2.02, 95% CI 1.03–4.00, *p* = 0.042), and severe toxicity (OR 2.70, 95% CI 1.23–5.92, *p* = 0.013)	Jennings et al. (2013)
rs11479; rs470119	QUASAR2 trial (ISRCTN45133151)	Grade 0–2 vs. grade 3+ adverse events	No association found at the threshold set in the study	Rosmarin et al. (2014)
	927 post-operative stage II/III colorectal cancer patients administered CAP in monotherapy or in combination regimens; rs470119 genotyped in 927 patients and rs11479 in 857 patients			
Meta-analysis: rs11479; rs470119; rs131804				


92 *TYMP* variants	940 post-operative stage II/III colorectal cancer patients administered CAP	Grade 0–2 vs. 3–4 HFS, diarrhea, and overall toxicity	No association found at the threshold set in the study	Rosmarin et al. (2015)
rs11479; rs131804; rs470119	130 breast and colorectal cancer patients administered CAP	Grade 3 HFS	No association found	Caronia et al. (2011)


rs11479; rs470119; rs131804	111 HER-2/neu- negative metastatic breast cancer patients administered CAP (50 control patients +61 experimental)	Grade 3–4 toxicity	No association found	Martin et al. (2015)


rs11479	185 gastric cancer patients administered CAP in combination regimens	Toxicities (hematological, gastrointestinal, HFS)	No association found	Meulendijks et al. (2017)
Sequencing of coding and exon-flanking regions of *TYMP*	Discover subset: 24 different cancer-type patients with grade ≥ 3 toxicity (without *DPYD*-risk variants or comedication), and 24 matched controls all administered CAP in monotherapy or in combination regimens	Grade 0–1, 2, 3–4 adverse events (HFS, diarrhea), overall toxicity (grade 0–1 vs. 2 vs. 3–4)	No association found	Hamzic et al. (2017)
rs470119	301 colorectal cancer patients administered CAP in monotherapy or in combination regimens	Grade ≥ 2 adverse events, overall toxicity, treatment administration delay	No association found	Pellicer et al. (2017)
rs11479	208 cancer patients administered CAP or placebo once daily	Grade ≥ 2 HFS	No association found	Yap et al. (2017)
rs470119	338 Chinese colorectal and gastric cancer administered FPs	Hematological, liver, gastrointestinal toxicity, and HFS	No association found	Liu et al. (2021)
rs11479	216 Brazilian gastrointestinal (*n* = 92) or colorectal cancer (*n* = 124) patients administered 5-FU in monotherapy or in combination regimens	Grade 1–4 and overall toxicity	No association found	Fernandes et al. (2021)

*CDA*, cytidine deaminase; CAP, capecitabine; HFS, hand and foot syndrome; OR, odds ratio; ADRs, adverse drug reactions; HER2, human epidermal growth factor receptor 2; 5-FU, 5-fluoruracil.

Jennings et al. (2013) were the first to report an association between *TYMP* rs11479 polymorphism and FP-induced ADRs, in an observational study including 253 colorectal cancer patients. Study endpoints included severe toxicity incidence and early dose modifications. Rs11479 minor allele was associated with both overall severe toxicity (OR 2.70, *p* = 0.013) and a need for early dose modifications (OR 2.02, *p* = 0.042). Results of this seminal study, however, were not replicated in other studies (Caronia et al., 2011; Rosmarin et al., 2014; Martin et al., 2015; Rosmarin et al., 2015; Hamzic et al., 2017; Meulendijks et al., 2017; Pellicer et al., 2017; Yap et al., 2017; Fernandes et al., 2021; Liu et al., 2021). Overall, it appears unlikely that *TYMP* polymorphisms could have a significant effect on CAP-induced toxicity.

#### 3.3.4 CYP2A6

Tegafur is an oral prodrug of 5-FU, predominantly administered in combination with uracil or as a combination fluoropyrimidine regimen, called S-1, consisting of tegafur, 5-chloro-2,4-dihydroxypyridine (CDHP), and potassium oxonate (Oxo) (Choi et al., 2012). Tegafur is converted to 5-FU by the cytochrome P450 isoenzyme CYP2A6. *CYP2A6* is a highly polymorphic gene and its genetic variations have been previously described in detail (Tanner and Tyndale, 2017). In brief, clinically significant alleles include CYP2A6*4 that leads to *CYP2A6* gene deletion, and several reduced activity alleles, including *2 and *5 to *35. There are significant differences in *CYP2A6* allele frequency among ethnicities; a higher prevalence of *CYP2A6* defective alleles in Asian and African American populations has been reported. Additionally, CYP2A6 is a highly inducible enzyme. Among CYP2A6 inducers, estrogens may account for gender related differences in CYP2A6 activity (Tanner and Tyndale, 2017).

The presence of *CYP2A6* reduced activity alleles has been extensively studied in association with tegafur pharmacokinetics (Table 6). It appears that CYP2A6 poor metabolizer phenotype leads to lower 5-FU AUC, lower tegafur oral clearance, and higher tegafur C_max_ and AUC (van Schaik, 2005; Kaida et al., 2008; Hirose et al., 2010; Ishii et al., 2010; Kim et al., 2011; Kim et al., 2016). It can be expected therefore that CYP2A6 poor metabolizers are poor responders to tegafur. Several studies have shown that carriers of *CYP2A6* wild-type alleles present with higher response rates to tegafur or S-1 (Kong et al., 2009; Park et al., 2011; Kim et al., 2013; Jeong et al., 2017; Yang et al., 2017; Kim et al., 2018). Based on pharmacokinetic data, the relative risk for tegafur-induced toxicity may be decreased for CYP2A6 poor metabolizers. Indeed, the majority of the studies that assessed the effect of *CYP2A6* defective alleles on tegafur-induced toxicity did not find an association (Kaida et al., 2008; Kong et al., 2009; Hirose et al., 2010; Ishii et al., 2010; Kim et al., 2011; Park et al., 2011; Choi et al., 2012; Fang et al., 2012; Kim et al., 2013; Kim et al., 2016; He et al., 2017; Jeong et al., 2017; Kim et al., 2018) (Table 6). Some sparse associations that have been published for common *CYP2A6* defective alleles did not remain significant in multivariate analyses (Tsunoda et al., 2011; Kim et al., 2017) and the authors suggest that *CYP2A6* defective alleles are not involved in tegafur-induced toxicity. However, it cannot be excluded that toxicity in CYP2A6 poor metabolizers may occur due to alternative tegafur-activating pathways. Exome sequencing of *CYP2A6* has revealed additional variants, such as rs60823196 and rs138978736. For these variants, an association with grade 3–4 diarrhea was shown in 60 patients treated with S-1 plus oxaliplatin (OR 6.43 for rs60823196 and OR 14.86 for rs138978736, respectively), however, their functional effect on CYP2A6 is still unknown (Yang et al., 2017). *CYP2A6* variations therefore could be considered as an additional genetic factor to guide the choice of FP therapy leading to avoidance of tegafur-based schemes in carriers of *CYP2A6* defective alleles.

**TABLE 6:** Studies examining the effect of *CYP2A6* gene polymorphisms on tegafur pharmacokinetics and response.

Examined *CYP2A6* variants	Study population	Endpoints	Results	Ref
CYP2A6*1; CYP2A6*4C; CYP2A6*7; CYP2A6*9	46 Japanese patients with non-small-cell lung cancer administered S-1 in combination with cisplatin (*n* = 31) or in monotherapy (*n* = 15)	Tegafur and 5-FU pharmacokinetics: AUC_0–10hr_, C_max_, T_max_	CYP2A6*4C carriers have lower 5-FU C_max_, lower 5-FU AUC_0-10_ and higher tegafur AUC_0-10 _in No association with toxicity endpoints	Kaida et al. (2008)
	Patients grouped into CYP2A6*4C carriers (*n* = 31) and non-carriers (*n* = 15)	Efficacy endpoints: response rate, adverse events		
CYP2A6*1; CYP2A6*4A; CYP2A6*5; CYP2A6*7; CYP2A6*9; CYP2A6*10; CYP2A6*11	34 patients with progressive/recurrent digestive organ cancer administered S-1	Pharmacokinetic endpoints: plasma 5-FU concentration	Lower tegafur, and higher 5-FU concentration in the carriers of one variant allele compared to the carriers of two variant alleles (*p* = 0.0309 and *p* = 0.0025, respectively)	Ishii et al. (2010)
		Efficacy endpoints: antitumor effect, adverse events	No association with efficacy endpoints	
CYP2A6*1; CYP2A6*4; CYP2A6*7; CYP2A6*9; CYP2A6*10	43 Asian patients with locally advanced gastric cancer administered S-1 in combination with docetaxel	Pharmacokinetic parameters: tegafur, 5-FU CDHP AUC_0–12hr_, C_max_	Carriage of two variant alleles associated with higher mean C_max_ for tegafur (*p* = 0.045) and worse survival	Kim et al. (2016)
		Efficacy endpoints; response rate, PFS, OS		
		Adverse events		
CYP2A6*1; CYP2A6*4; CYP2A6*7; CYP2A6*9	57 Japanese patients with metastatic or recurrent solid malignancies administered S-1	Pharmacokinetic parameters; tegafur, 5′-FU, and CDHP AUC_0–24hr_, C_max_, T_1/2_, CL	Carriers of two variant alleles had 58% less tegafur CL compared to wild-type patients	Hirose et al. (2010)
		Adverse events		
CYP2A6*1; CYP2A6*4; CYP2A6*7; CYP2A6*9; CYP2A6*10	49 Korean patients with recurrent/metastatic adenocarcinoma of the biliary tract administered S-1 in combination with oxaliplatin	Pharmacokinetic parameters; tegafur, 5-FU AUC_0–24hr_ C_max_, T_max_ Efficacy endpoints; response rate, PFS, OS	5-FU concentrations tended to be higher and tegafur’s lower in the homozygous for the wild type allele patients compared to those carrying variant alleles	Kim et al. (2011)
		Adverse events	No association found for toxicity or efficacy endpoints	
CYP2A6*1; CYP2A6*4; CYP2A6*7; CYP2A6*9; CYP2A6*10	50 metastatic gastric cancer Asian patients administered S-1 in combination with docetaxel	Response rate, PFS, OS, adverse events	Carriers of at least one wild type allele had higher response rate and PFS than the homozygous for variant alleles both in univariate and multivariate analysis (*p* = 0.04 and *p* = 0.001, respectively)	Kong et al. (2009)
			No association with toxicity endpoints	
CYP2A6*4; CYP2A6*7; CYP2A6*9; CYP2A6*10	106 patients with metastatic gastric cancer administered S-1 in combination with cisplatin	Response rate, PFS, and OS	CYP2A6*4 associated with lower response rate	Park et al. (2011)
		Adverse events	(OR 0.220, 95% CI 0.067–0.719, *p* = 0.012), shorter PFS (OR 2.288, 95% CI 1.245–4.207, *p* = 0.008), and shorter OS (OR 3.118, 95% CI 1.483–6.558, *p* = 0.003)	
			No association with toxicity endpoints	
CYP2A6*4; CYP2A6*7; CYP2A6*9	42 patients with unresectable or metastatic adenocarcinoma of the colon or rectum administered S-1 in combination with irinotecan and oxaliplatin	Response rate, PFS, OS, adverse events	Carriers of the variant alleles were associated with poorer response rate (*p* = 0.05)	Kim et al. (2013)
			No association with toxicity endpoints	
CYP2A6*1; CYP2A6*4; CYP2A6*7; CYP2A6*8; CYP2A6*9; CYP2A6*10	200 Asian gastric cancer patients administered S-1	RFS, OS, adverse events	Carriers of variant alleles had poorer RFS (HR 3.41, 95% CI 1.01–11.52, *p* = 0.049 for heterozygous, HR = 3.41, 95% CI 1.16–13.93, *p* = 0.028 for homozygous)	Jeong et al. (2017)
			No association with toxicity endpoints	
Whole exome sequencing of *CYP2A6*, 22 SNPs identified, among them	60 gastric cancer patients administered SOX (oxaliplatin + S-1)	PFS, OS, adverse events	rs60823196 and rs138978736 associated with grade 3–4 diarrhea (OR 4.905, 95% CI 1.38–17.45, *p* = 0.02 and OR 15.860, 95% CI 4.05–62.11, *p* = 0.0002, respectively)	Yang et al. (2017)
CYP2A6*5; CYP2A6*7; CYP2A6*8; CYP2A6*10; CYP2A6*11; rs60823196; rs138978736; rs150586234; rs771265125; rs58571639; rs2644907; rs60988093			rs138978736 associated with shorter OS (*p* = 0.006) in the subgroup of 30 patients administered S-1 as adjuvant chemotherapy	
CYP2A6*1; CYP2A6*4; CYP2A6*7; CYP2A6*8; CYP2A6*9; CYP2A6*10	Elderly patients with recurrent or metastatic gastric cancer randomised in CAP-treated (*n* = 53) and S-1-treated (*n* = 52) groups	Response rate, disease control rate, PFS, OS, adverse events	In the S-1 subgroup, patients carrying two variant alleles (except for *8) had shorter PFS (HR 2.46, 95% CI 1.20–5.05, *p* = 0.015) and OS (HR 2.22, 95% CI 1.14–4.31, *p* = 0.019)	Kim et al. (2018)
			No association for toxicity endpoints	
CYP2A6*1A; CYP2A6*1B; CYP2A6*4C	77 Chinese patients with cancer of the digestive system administered S-1	Response rate, disease progression, adverse events	No association found	Fang et al. (2012)
CYP2A6*4; CYP2A6*7; CYP2A6*9; CYP2A6*10	29 patients with metastatic or recurrent colorectal adenocarcinoma administered S-1 in combination	Response rate, survival, adverse events	No association found	Choi et al. (2012)
CYP2A6*1D; CYP2A6*4; CYP2A6*7; CYP2A6*9	99 stage II-III colorectal cancer patients administered tegafur-uracil in combination with leucovorin	Grade ≥2 toxicity, overall toxicity	Variant alleles associated with hyperbilirubinemia in univariate analysis (*p* = 0.039)	Tsunoda et al. (2011)
CYP2A6*4; CYP2A6*7; CYP2A6*9; CYP2A6*10	91 patients with confirmed adenocarcinoma of the rectum orally administered tegafur- uracil	Pathologic complete response, PFS, OS, adverse events	Variant alleles associated with grade 2 or higher leucopenia (*p* = 0.022) and stomatitis (*p* = 0.012)	Kim et al. (2017)

*CYP2A6*, cytochrome P450 2A6; 5-FU, 5-fluoruracil; AUC, area under the curve; C, concentration; PFS, progression free survival; OS, overall survival; CDPH, 5-chloro-2,4-dihydroxypyridine; CL, clearance; RFS, relapse free survival.

#### 3.3.5 Uridine monophosphate synthetase, *UMPS*

Orotate phosphoribosyltransferase (OPRT), also known as uridine monophosphate synthetase (UMPS), is responsible for 5-FU phosphorylation into its active metabolite FUMP (Wang et al., 2014). Studies assessing the effect of *UMPS* polymorphisms on FP therapeutic outcomes are presented in Table 7.

**TABLE 7:** Studies examining the effect of *UMPS* gene polymorphisms on FP-induced adverse events.

Examined *UMPS* variants	Study population	Endpoints	Results	Ref
rs1801019	69 primary colon or rectum carcinoma patients administered bolus 5-FU in combination with leucovorin	Grade 3–4 neutropenia, and diarrhea, time to onset of toxicity	rs1801019 minor allele	Ichikawa et al. (2006)
			associated with grade 3–4 neutropenia (*p* = 0.0393), diarrhea (OR 13.3, 95% CI 1.9–280.9, *p* = 0.026) and earlier time to toxicity onset (*p* < 0.0001)	
rs1801019	99 stage II-III colorectal cancer patients administered tegafur-uracil in combination with leucovorin	Grade ≥2 toxicity, overall toxicity	rs1801019 minor allele associated with grade 3 diarrhea (*p* = 0.031), grade 2–3 anorexia (*p* = 0.035), hyperbilirubinemia (*p* = 0.013) time to severe toxicity onset (*p* < 0.0002) In multivariate analysis: rs1801019 homozygous for the minor allele associated with grade 3 diarrhea (OR 19.84, 95% CI 1.82–215.90, *p* = 0.014) and overall toxicity (OR 17.60, 95% CI 1.58–195.89, *p* = 0.020)	Tsunoda et al. (2011)
rs1801019	91 patients with confirmed adenocarcinoma of the rectum orally administered tegafur- uracil	Toxicity	rs1801019 associated with grade ≥2 diarrhea (*p* = 0.018) and modesty associated with grade ≥2 abdominal pain (*p* = 0.067)	Kim et al. (2017)
rs2279199; rs4678145; rs1139538; rs9844948; rs3772804	301 colorectal cancer patients administered CAP in monotherapy or in combination regimens	Grade ≥ 2 adverse events, overall toxicity, treatment administration delay	rs2279199 protective for grade >2 nausea/vomiting (OR 0.210, 95% CI 0.049–0.900, *p* = 0.036)	Pellicer et al. (2017)
			rs4678145 associated with grade >2 fatigue (OR 4.542, 95% CI 1.557–13.243, *p* = 0.006)	
34 *UMPS* SNPs	940 post-operative stage II/III	Grade 0–2 vs. 3–4 HFS, diarrhea, overall toxicity	No association found at the threshold set in the study	Rosmarin et al. (2015)
	colorectal cancer			
	patients			
	administered CAP			
rs1801019, Meta-analysis: rs1801019; rs3772809	QUASAR2 trial (ISRCTN45133151) 927 post-operative stage II/III colorectal cancer patients administered CAP in monotherapy or in combination regimens	Grade 0–2 vs. grade 3+ adverse events	No association found at the threshold set in the study	Rosmarin et al. (2014)


rs1801019; rs2279199; rs4678145	338 Chinese colorectal and gastric cancer administered FPs	Hematological, liver and gastrointestinal toxicity, HFS	No association found	Liu et al. (2021)
rs1801019	253 colorectal cancer patients administered CAP (*n* = 159) or 5-FU (*n* = 94) in monotherapy or in combination regimens	Early dose modifications (delays or reductions), severe toxicity	No association found	Jennings et al. (2013)
rs1801019	216 Brazilian gastrointestinal (*n* = 92) or colorectal cancer (*n* = 124) patients administered 5-FU in monotherapy or in combination regimens	Grade 1–4 toxicity	No association found	Fernandes et al. (2021)
rs1801019; rs3772809	Exploration and validation cohort administered 5-FU in combination regimens (160 and 340 patients, respectively)	Gastrointestinal toxicity (stomatitis/pharyngitis, nausea/vomiting, diarrhea)	No association found	Afzal et al. (2011)

rs1801019	93 gastric cancer patients treated with FP-based chemotherapy	Adverse events	No association found	Cordova-Delgado et al. (2021)

*UMPS*, uridine monophosphate synthetase; OR, odds ratio; CAP, capecitabine; HFS, hand and foot syndrome; FPs, fluoropyrimidines; 5-FU, 5-fluoruracil.

In the seminal study that assessed the potential association of *UMPS* polymorphisms with FP-toxicity, Ichikawa et al. (2006) reported a significant association between *UMPS* 638G>A variant (rs1801019) and 5-FU induced grade 3–4 toxicity in a cohort consisting of 69 5-FU treated colon or rectum cancer patients. The missense *UMPS* 638G>A variant was associated with increased risk for severe neutropenia (*p* = 0.039), diarrhea (OR 13.3, *p* < 0.0001) and an earlier onset of toxicity (*p* < 0.0001) in carriers of the minor allele. These results were replicated by Tsunoda et al. (2011) in a cohort of 99 colorectal cancer patients treated with tegafur-uracil. In this study, *UMPS* 638G>A variant was associated with grade 3 toxicity (OR 17.60, *p* = 0.02), grade 3 diarrhea (*p* = 0.031), grade 2–3 anorexia (*p* = 0.035), hyperbilirubinemia (*p* = 0.013) and a shorter time of toxicity onset (*p* < 0.0002) (Tsunoda et al., 2011). The association of the multiallelic 638G>C allele with diarrhea was also replicated in another study on 91 tegafur-treated rectum cancer patients (*p* = 0.018) (Kim et al., 2017). Pellicer et al. (2017) reported the association of two additional *UMPS* variants, rs2279199 and rs4678145, with nausea/vomiting (OR 0.21, *p* = 0.036) and fatigue (OR 4.54, *p* = 0.006), respectively.

These results are really promising in identifying an additional genetic marker for FP-induced ADRs that can be included in a polygenic dosing algorithm. However, several studies did not identify an association of *UMPS* variants with FP-response (Afzal et al., 2011; Jennings et al., 2013; Rosmarin et al., 2014; Cordova-Delgado et al., 2021; Fernandes et al., 2021; Liu et al., 2021). Therefore, *UMPS* polymorphisms in clinical decisions on FPs is still under investigation.

#### 3.3.6 Other genes in FP metabolic pathway

Several studies examined the potential significance of additional, less investigated genes, encoding enzymes implicated in the metabolic or catabolic pathway of FPs. These enzymes are dihydropyrimidinase (DPYS) and β-ureidopropionase 1 (UPB1) participating in 5-FU excretion pathway (Lam et al., 2016) and the regulatory component of ribonucleotide reductase (RR), encoded by *RRM1*, that is involved in 5-FU metabolism to active metabolites (Aoki et al., 2013). Results of FP pharmacogenomic studies on these genes are shown in Table 8.

**TABLE 8:** Studies examining the effect of *DPYS*, *UPB1* and *RRM1* gene variants on FP-induced adverse events.

Examined variants	Study population	Endpoints	Results	Ref
Sequencing of entire coding sequence and flanking intronic regions	113 cancer patients (67 with grade 3–4 toxicity) treated with FPs and 69 non-cancer individuals as control group	Grade 3–4 hematological toxicity	Homozygous -1CC patients were at increased risk for mucositis (OR 4.13, 95% CI 1.51–11.31, *p* = 0.006), diarrhea (OR 2.12, 95% CI 0.94–4.76, *p* = 0.007) and gastrointestinal toxicity (OR 3.54, 95% CI 1.59–7.88, *p* = 0.002)	Fidlerova et al. (2010)
*DPYS*			*DPYS* −1C was more frequent in the high-toxicity group (66%) compared to the well-tolerance-group (57%) and healthy individuals (54%), *p* = 0.06	
−1 T>C; −58 T>C			−58C allele was rarer in the high-toxicity group (47%) compared to the well-tolerance-group (59%) and the non-cancerous individuals (57%), *p* = 0.02	
			−58C allele was associated with lower risk for overall gastrointestinal toxicity (OR 0.40, 95% CI 0.17–0.93, *p* = 0.03) and leukopenia (OR 0.29, 95% CI 0.08–1.01, *p* = 0.05)	
*DPYS* rs61758444; rs36027551; rs34895123; rs2669429	430 FP-treated patients with gastrointestinal cancer	Adverse events, overall toxicity	No association found	Loganayagam et al. (2013)
*DPYS* rs2669429	338 Chinese colorectal and gastric cancer administered FPs	Hematological, liver and gastrointestinal toxicity, HFS	No association found	Liu et al. (2021)
Sequencing of entire coding sequence and flanking intronic regions	113 different cancer type patients (67 with grade 3–4 toxicity) treated with FPs and 69 individuals as control group	Grade 3–4 hematological toxicity and/or gastrointestinal toxicity	−80 GG patients were at risk for grade 3–4 mucositis (OR 7.5, 95% CI 2.60–21.60, *p* = 0.0002) and at decreased risk for severe diarrhea (OR 0.379, 95% CI 0.15–0.98, *p* = 0.044)	Fidlerova et al. (2012)
*UPB1*				
−80 C>G; −17 A>T; 105–61 A>G				


*DPYS*	514 cancer patients administered 5-FU or CAP regimens (164 in the discovery cohort, 85 patients with severe toxicity, 79 matched controls)	Overall toxicity	Several associations present for *DPYS* −1C, −58C and *UPB1* −80G alleles and overall toxicity, gastrointestinal toxicity and nausea	Kummer et al. (2015)
−1 T>C; −58 T>C			When adjusted for *DPYD* variants:	
*UPB1* −80 C>G			*DPYS -*1C and *UPB1* –80G associated with FP toxicity (OR 1.78, 95% CI 1.03–3.08, *p* = 0.039 and OR 1.77, 95% CI 1.08–2.92, *p* = 0.047, respectively)	
			For *DPYS*, results potentially driven by 5-FU group	

69 *DPYS* variants; 30 *UPB1* variants; 29 *RRM1* variants	940 post-operative stage II/III	Grade 0–2 vs. 3–4 HFS, diarrhea, and overall toxicity	No association found at the threshold set in the study	Rosmarin et al. (2015)
	colorectal cancer			
	patients			
	administered CAP			
*RRM1* rs12806698; rs1042927	216 Brazilian gastrointestinal (*n* = 92) or colorectal cancer (*n* = 124) patients administered 5-FU in monotherapy or in combination regimens	Grade 1–4 adverse events, overall toxicity	No association found	Fernandes et al. (2021)


*DPYS,* dihydropyrimidinase; *UPB1*, β-ureidopropionase 1; *RRM1*, regulatory component of ribonucleotide reductase; FPs, fluoropyrimidines; OR, odds ratio; CAP, capecitabine; 5-FU, 5-fluoruracil; HFS, hand and foot syndrome.

Fidlerova et al. (2010) were the first to analyze genes downstream of DPD. In two sequential studies, hematological and gastrointestinal toxicity rates were analyzed within *DPYS* and *UPB1* genotypes in a cohort of 113 cancer patients (67 of whom experienced severe toxicity, and 46 with good tolerance to FP) and 69 non-cancer individuals. *DPYS* −1CC genotype was more frequent within the patient group who experienced diarrhea (OR 2.12, *p* = 0.007), gastrointestinal toxicity (OR 3.54, *p* = 0.002), and severe mucositis (OR 4.13, *p* = 0.006), whereas −58C allele was associated with a lower risk for overall gastrointestinal toxicity (OR 0.4, *p* = 0.03) and leucopenia (OR 0.29, *p* = 0.05) (Fidlerova et al., 2010). Similar results for *DPYS* −1C were reported in a study including 514 patients; *DPYS* -1C allele was associated with overall toxicity (OR 1.78, *p* = 0.039), gastrointestinal toxicity (OR 3.06, *p* = 0.006) and nausea (OR 4.46, *p* = 0.016), while −58C had a protective effect against gastrointestinal toxicity (OR 0.55, *p* = 0.024) and nausea (OR 0.45, *p* = 0.014) (Kummer et al., 2015). Interestingly, those associations were stronger in patients receiving 5-FU based regimens.

For *UPB1*, Fidlerova et al. (2012) have found that −80C>G variant minor allele was an independent predictor of severe mucositis (OR 7.5, *p* = 0.0002). Additionally, a borderline association with leukopenia (*p* = 0.076) and hematologic toxicities (*p* = 0.061) has been reported for *UPB1* −80C>G allele (Kummer et al., 2015). For *RRM1* variants, no association with FP response or toxicity has been found (Fernandes et al., 2021).

In conclusion, *DPYS* and *UPB1* appear as attractive candidates of FP variable response. The possibility of their inclusion in a polygenic dosing algorithm is worthy of further consideration.

#### 3.3.7 Genes affecting response to FP-based chemotherapeutic schemes

FPs can be administered as monotherapy or in combination with other chemotherapeutics such as oxaliplatin (FOLFOX, XELOX), irinotecan (FOLFILIRI, XELIRI), taxanes (paclitaxel, docetaxel), and monoclonal antibodies. In chemotherapeutic combination schemes, incidence of ADRs or treatment efficiency can be induced by other drugs beyond FPs. Therefore, in patients treated with FP-including chemotherapeutic schemes, additionally to polymorphisms of genes encoding enzymes involved in FP pathway, variations in genes encoding membrane transporters (De Mattia et al., 2015), nuclear receptors (De Mattia et al., 2013; Cecchin et al., 2016; De Mattia et al., 2018; De Mattia et al., 2019a), transcription factors and molecules mediating downstream therapeutic pathways (De Mattia et al., 2021) may be associated with therapy resistance or ADR incidence.

Currently, established pharmacogenomic markers for chemotherapeutic regimens including irinotecan exist; both FDA and EMA suggest dose reductions for the homozygous carriers of UGT1A1*28 allele (FDA, 2015b; EMA, 2015). Other gene associations include variations in the genes coding for rate-limiting enzymes of the nucleotide excision DNA repair system *ERCC1* and *ERCC2* in association with response and survival to oxaliplatin (Di Francia et al., 2013; De Mattia et al., 2015). *CYP3A4*, *CYP3A5*, and *CYP1B1* genetic variants as well as variations in *ABCB1* transporter have been associated with response to taxanes (De Iuliis et al., 2015).

The specific review concentrates on the idea of a potential polygenic dosing algorithm for FPs, however, precision medicine in oncology relies on onco-omics (Ragia and Manolopoulos, 2022), thus, a broad spectrum of genes should be implemented to predict response to combination chemotherapeutic schemes.

## 4 A polygenic algorithm for FP dosing: New challenges in oncology

FP pharmacogenomics has entered clinical practice via the well-documented association of *DPYD* with FP-induced severe toxicity, there is still, however, an unmet medical need to predict and reduce severe FP-induced toxicity in cancer patients receiving chemotherapy. We anticipate that eventually there will be advances in FP genotype-based clinical decisions in oncology. Firstly, we anticipate that *DPYD* will be globally accepted as for its impact on FP dose requirements. Though in Europe EMA has endorsed *DPYD* genotyping prior to FP administration, FDA is still rather modest on the modifications of drug label. Most likely, *DPYD* will still be at the center of attention regarding FP pharmacogenomics since there is still much to understand on the clinical significance of *DPYD* rare variants, on the impact of the identified variants on FP dosing and toxicity in different populations, and on the optimal ethnicity-based variant combination for preemptive genotyping. *DPYD* is on the prime time due to its dominant role in 5-FU metabolism, irrespectively of the administered FP. As important as this step for FP pharmacogenomics may be, we suggest that this may not be the whole story but just the beginning of it. We believe, therefore, that *DPYD* will not remain for much longer as the sole genetic factor affecting FP dosing decisions.

Polygenic scores are primarily been applied for disease risk prediction, but they have yet to be broadly adopted in the field of pharmacogenomics. Drug dosing requirements based on a multigenic model are applied for vitamin K antagonists and this consists the most well characterized example of the application of a pharmacogenomic dosing algorithm in clinical practice. Hitherto, in oncology, such an approach has not been developed, however, the majority of pharmacogenomic drug safety studies have been conducted in antineoplastics (Siemens et al., 2022) suggesting that we are approaching to the era of a FP polygenic dosing algorithm.

Towards this direction, accumulated evidence shows that *TYMS* and *ENOSF1* low expression variants are strongly associated with FP toxicity and can improve the *DPYD*-pharmacogenomic guided dosing. Therefore, *DPYD*, *TYMS* and *ENOSF1* can form the core of a polygenic algorithm. In patients with normal DPD activity, *TYMS* and *ENOSF1* polymorphisms may guide the appropriate dose reductions. For *ENOSF1* rs2612091 it has been recently shown that when it is integrated to the *DPYD*-based prediction model for FP-induced toxicity, it significantly improves prediction of global toxicity, hematological toxicity, HFS, and diarrhea (Palles et al., 2021). These results, therefore, support our idea and we anticipate that more studies will move from single *DPYD* based FP-dosing to a multigenic dosing approach.

Depending on the FP used, additional gene variations can improve the outcome and safety of therapy. For CAP, it appears that multiple genes associated with reduced CAP catabolism can help in predicting HFS. Following liver metabolism, CAP is further catabolized to 5-FU by the enzymes encoded by *CES2/1, CDA* and *TYMP*. Polymorphisms in these genes have been associated with CAP-induced HFS. For *CES1* and *CDA* a more prominent association appears, suggesting that they merit further investigation for their contribution to reducing CAP-induced toxicity and overall incidence of ADRs. For the second 5-FU prodrug, tegafur, CYP2A6 is crucial for its bioactivation. *CYP2A6* defective alleles seriously impact tegafur pharmacokinetics and 5-FU generation. CYP2A6 poor metabolizers can potentially benefit from alternative treatment.

Downstream enzymes that may affect FP toxicity include UMPS, DPYS, UPB1, and RR participating either in 5-FU phosphorylation into its active metabolites or in 5-FU excretion. These genes are relatively less studied, however, several promising associations with increased risk for FP toxicity have been reported. Currently, there is no clear role for these genes in a FP polygenic algorithm, however, in the case of FP-induced toxicity in patients who do not carry any other variation, *UMPS*, *DPYS*, *UPB1,* and *RR* genotyping may help in elucidating rare cases of toxicity.

Therapeutic response to FP involves several pharmacodynamic aspects. Though pharmacodynamic genes downstream to FP active nucleotides are not expected to strongly affect FP dose requirements, it should be acknowledged that enzymes involved in DNA repair, cell cycle and apoptosis may drive tumor resistance to chemotherapy and in synergy with pharmacokinetic genes may increase risk for ADRs (Tecza et al., 2018; Boige et al., 2019; Varma et al., 2020).

Beyond the effect of single genetic variations to FP-induced toxicity and dose requirements, several other hurdles exist until a polygenic dosing algorithm could be applied for FPs. Firstly, for most genes, an increased number of variations has been identified; the role of these variations in enzyme activity has yet to be determined. In addition, the existence of additional deleterious variants, albeit rare in frequency, cannot be excluded. Consequently, the current panel of variations is rather broad, potentially affecting the sensitivity of such an approach. To overcome this limitation, advances in genotyping technologies can be helpful. The process of building a FP polygenic dosing algorithm will benefit from (very) high throughput genotyping platforms, in multiple ways. Next-generation sequencing (NGS) technology can be used both to screen the genetic variations in all pharmacogenes of an individual, as well as to identify rare variants in pharmacogenes, thus adding important information to predict drug response (Ingelman-Sundberg et al., 2018; Tafazoli et al., 2021). It should be acknowledged that rare variants in pharmacogenes can shed light on toxicity mechanisms and have emerged as clinically useful markers in predicting ADRs; not only they contribute significantly to pharmacogenomic variability, but also their frequency varies within populations of different ethnicity (Ingelman-Sundberg et al., 2018; Runcharoen et al., 2021). Recently, it was shown that polygenic risk scores adjusted for common variants can efficiently improve power in rare variant association discovery (Jurgens et al., 2023). Therefore, a polygenic dosing algorithm might help in the identification of additional pathways associated with FP response.

Ethnicity could be a major factor that should be taken into account for the selection of genetic variations. It has been shown for different pharmacogenomic applications that even for common variations within established pharmacogenes, current dosing algorithms should be adjusted per ethnicity. Such an example is the *CYP2C9*/*VKORC1* pharmacogenomic dosing algorithm for vitamin K antagonists (Ragia et al., 2017b). For FPs, it appears that the frequency of pharmacogene polymorphisms varies within populations of different ancestry; in Asians, *DPYD* variations have only a minor role in FP-related toxicity (Kanai et al., 2022), whereas a higher prevalence of *CYP2A6* defective alleles has been reported (Tanner and Tyndale, 2017). Therefore, we expect that different variants will be incorporated in a FP polygenic algorithm and that, in each case, the predictive value of the algorithm should be tested and validated within populations. Moreover, even though a 100% overall concordance has been shown for *DPYD* genotyping in blood and tissue samples (Morelli et al., 2022), it should be considered that in tumor tissue genetic alterations are possible in other FP-associated genes that may affect tissue-specific FP catabolism and action. Additionally, the expression of genes regulating FP bioavailability can be influenced by transcriptional factors activated by the tumor-related characteristics (Wu et al., 2016; Suenaga et al., 2021; Delhorme et al., 2022). Therefore, genetic variation of these factors might also play a role in FP dose requirements.

In pharmacogenomics, multiple interactions exist within genes and it cannot be excluded that several genetic associations are masked (Ragia et al., 2014) raising thus the need for analyses in sub-phenotypes (Ragia et al., 2017a). In cancer, gene interactions are expected in different layers, not only within pharmacogenes, but additionally within cancer predisposing genes (Capellini et al., 2021). Additionally, gene*environment interactions exist both in cancer development (Mbemi et al., 2020) and in response to chemotherapy (Cui et al., 2017); once such interactions are identified, they can be implemented in therapeutic strategies. More importantly, in cancer, gender interactions are well established; for 5-FU, it has been reported that women are *a priori* at increased ADR risk due to reduced drug elimination (Mueller et al., 2013). Additionally, gender-related biological factors can influence the (pharmaco)genetic associations (Mezzalira and Toffoli, 2021). The pharmacogenomic impact of gene*gender interactions is currently less studied, however, data previously published indicate that in FP response pharmacogenomic associations in genes beyond *DPYD* are gender dependent (Ioannou et al., 2021, 2022). In a recent research article published by our team, a gender dependent association of low *TYMS* expression alleles has been found: *TYMS*-TSER 2R/2R genotype was associated with FP dose reduction due to ADRs in female patients, a finding potentially attributed to estrogen receptor regulation of TS expression (Ioannou et al., 2021). Similarly, we have found a gender*MTHFR interaction possibly interfering with FP response: in gender stratification analysis *MTHFR* -677C>T polymorphism increased both need for FP dose reduction (OR 5.05) and percentage of dose reduction (*β* = 3.318) in female patients, whereas no differences were present in pooled sample analyses (Ioannou et al., 2022). This gender dependent association is potentially driven by variable homocysteine levels between two genders and merits further investigation. Thereby, when considering a polygenic algorithm for FP-dosing, gene*gender interactions should be taken into account.

Apart from pharmacogenomics, the underlying factors predisposing patients to FP-induced toxicity are largely unknown. However, several patient-related factors exist that increase risk for FP toxicity. Advanced age is an independent predictor of severe toxicity and elderly patients should be closely monitored for FP-induced ADRs (Stein et al., 1995). Pre-existing diseases, such as cardiovascular diseases (CVD), hepatic impairment, and renal insufficiency, as well as CVD risk factors (hypertension, hyperlipidemia and smoking), have been studied as for their association with increased risk for 5-FU-induced cardiotoxicity (Brutcher et al., 2018; Sara et al., 2018). The perception of incorporating non-genetic factors into polygenic risk score models is currently being evaluated for disease risk assessment (van Dam et al., 2023). This approach holds promise to increase the discriminative power of the often low variance explained solely by the genetic factors for the predictive outcome(s). We propose, therefore, that non-genetic factors, once identified, could also be added in a FP-polygenic dosing algorithm to improve pharmacogenomic predictions.

Heterogeneity exists not only in cancer tissue genetics and in cancer stratification but also in ADR symptoms. Pharmacogenomics of FPs currently involves cohorts of patients with different solid tumors, treated often with a combination of FP-based scheme, and ADRs on different systems are grouped together to increase statistical power. Pooled analyses can indeed highlight universal associations, however, other pharmacogenomic associations, cancer- or system-specific, may be underestimated. On the other hand, in schemes that include chemotherapeutic combinations, incidence of ADRs can be induced by other drugs beyond or additionally to FPs. Therefore, future research should focus on distinct phenotypes to reduce heterogeneity, as well as on integration of pharmacogenomics of every single administered drug.

We have herein discussed extensively the genetic architecture of FP-induced toxicity and the potential role of gene*gene, gene*gender, and gene*environment interactions in FP dosing decisions. In therapeutics, however, drug-drug interactions consist a major driver of drug-induced ADRs. In FP therapy, several clinically relevant drug-drug interactions, pharmaceutical, pharmacodynamic, and pharmacokinetic, exist, either among chemotherapeutics or with supportive care drugs that increase risk for FP-induced toxicity (van Leeuwen et al., 2011; Ramsdale et al., 2022; van Leeuwen et al., 2022). Precision medicine in oncology, thus, can also have a role in polypharmacy guidelines.

While we propose herein the concept of a polygenic FP dosing algorithm, the actual form and mathematical equation of such an algorithm is beyond the scope of the present work. Several tools exist to guide this procedure. Machine learning could be of help in prioritizing the genetic variants included in the algorithm aiding to select the optimal regimen and doses, and, if employed, signatures with clinical utility can be constructed (Panagopoulou et al., 2021; Pandi et al., 2021). Artificial intelligence models have rapidly gained attention in pharmacological field. In pharmacogenomics, indicatively, studies published so far highlight the use of artificial intelligence models for warfarin dose prediction in Asian populations (Jahmunah et al., 2023) and antidepressant response (Bobo et al., 2022). Initial data for the use of machine learning and artificial intelligence in FP treatment are also available, focusing on *DPYD* variant classification (Shrestha et al., 2018) and on identification of genomic and transcriptomic biomarkers for 5-FU response (Kong et al., 2020). Therefore, a multi-omics integrative approach for FP-induced toxicity assisted by computational sciences can shed additional light on this complexity (Ragia and Manolopoulos, 2022).

In the present review we extensively discuss current pharmacogenomic evidence that can guide FP dosing. It should be acknowledged, however, that several hurdles exist at multiple levels until a polygenic dosing algorithm could conceivably be implemented in clinical practice. These include, the identification of the associations, the validation of the polygenic algorithm, the exact dosing recommendations, and their incorporation in routine clinical practice. Once a polygenic dosing algorithm is formed, prospective pharmacogenomic clinical trials will be required to assess whether this approach is indeed superior compared to standard dosing. Nevertheless, several limitations also exist in standard dosing procedures that currently take into account body surface area (BSA)-adjusted dosing. A large proportion of FP-treated patients (more than 50%) are in the under-exposure range while more than 10% fall in the toxicity ranges (Gamelin et al., 2008; Saam et al., 2011; Li et al., 2023). To avoid further underdosing when adding multicomponent risk variants for toxicity risk prediction, current dosing strategies should be clinically improved. Once standard dosing is improved, the predictive power of pharmacogenomic markers becomes even stronger. Implementation of preemptive FP pharmacogenomics needs the acceptance of such an approach by oncologists. Therefore, we believe that clinical implementation relies on consensus of experts from oncological societies and chemotherapy boards worldwide.

## 5 Conclusion

Precision medicine in oncology has rapidly expanded throughout the last 20 years and significant milestones have been reached towards translation in clinical practice of gene based therapeutic options. *DPYD*-based dosing approach is one of them, however, we suggest that it may be only the first step towards FP therapy personalization. The prognostic value of *DPYD* genotyping can stand for the opening of a new era in FP pharmacogenomics. It is time to accept the critical challenges and move from single gene approach to a polygenic dosing algorithm approach to succeed in the ultimate goal of optimal and safer chemotherapy in cancer patients.
